# Report of Surgical Cases in the Buffalo General Hospital

**Published:** 1863-03

**Authors:** J. F. Miner


ART. IV.—*Report of Surgical Cases in the Buffalo General Hospital.* By J. F. Miner, M. D.

It is proposed to report the cases under treatment during the last four months, and to give in detail some of the more interesting and important ones, grouping and considering in classes others, less rare and instructive. It may not be improper in this connection to observe, that surgical practice in the Buffalo General Hospital, more nearly approaches private surgical practice in its character and conditions, than in most hospitals in this country. This arises from the necessity of compensation, and general rejection of the ordinarily large number of old, hopeless, worn-out, incurable applicants, which go in most cases to swell the number, but add nothing to the real amount of surgical observation.

These patients are for the most part citizens of Buffalo, or neighboring towns, who resort hither, not so much for a home during the cold season, as for surgical operation or treatment. Accidental injuries, fractures, contusions, lacerations, and all other serious injuries, often receive here their first dressings. Under these circumstances as favorable results should be expected as in private surgical practice.

The following list of diseases and operations are copied from the hospital record, and it is proposed to consider them in their order, describing any peculiarities or features of marked clinical interest:

| | | | |
|---|---|---|---|
| Amputations after injury, | 2 | Paralysis, | 1 |
| " on account of disease, | 1 | Syphilis and Gonorrhœa, | 23 |
| Abscesses, | 2 | Orchitis, | 1 |
| Gun-shot wounds, | 3 | Affections of the eye, | 5 |
| Fractures, | 5 | Exsections after fracture, | 1 |
| Diseases of bones and joints, | 3 | Laceration, | 2 |
| Varicose veins, | 3 | Erysipelas, | 2 |
| Affections of the uterus, | 3 | Ulcers, | 6 |

*Amputations.*—The first two cases were private patients, taken to the hospital for convenience of operation. The 1st was for injury of the hand, requiring the amputation of one finger, and meriting no attention beyond a notice in the record. The 2d case was on account of most terrible and severe injury caused by the foot being caught in the scoups or bins of an elevator, while revolving rapidly, by which means the foot was torn completely off at the ankle joint, and then again about midway of the leg, drawing the tendons out from a great length, and lacerating the muscles in a most frightful manner. This patient was 45 years old, of intemperate habits, and lived some distance from the place of accident. His employer, after sending a messenger for me, had tied a handkerchief and cord tightly around the leg to prevent bleeding, and on my arrival a tourniquet was also immediately adjusted upon it. He was removed to the hospital, and amputation was attempted below the knee, but it was found upon dividing the muscles, that they were so greatly injured as to make amputation above the knee necessary. He rallied from the effects of the shock, operation, &c., and appeared very well for two days after, when it became evident that the result would be unfavorable, and he died on the morning of the fourth day. This was to be expected, since amputation of the thigh after injury of this nature, is almost always fatal under favorable circumstnnces, and with the loss of blood necessary after such accident, the severity of shock incident to it, and debility of system caused by intemperance, nothing but a fatal result could have been for a moment expected. Notwithstanding the hopelessness of the undertaking, nothing remained but to make some dressing, and it could not be properly done in any other way. Amputation was a necessity, though death after it, but not by any means from it, was almost certain.

This resembled in many, nearly in all respects, injury from cannon ball; the shock, loss of blood, effects upon the muscles and bones, indeed all the conditions of cannon-shot removal of foot and leg. McLeod, in his notes of the surgery of the Crimean war, says, that amputation of the upper third of the femur, after gun-shot injury, was fatal in all cases. Eighty per cent. of the cases were fatal when amputation was made at any point of the femur. Other statistics upon this subject, correspond very nearly, and none leave us anything to expect under the circumstances attending the case we have recited.

3d Case. Mr.———, aged 33, of light complexion, light hair, and scrofulous appearance, was admitted to the Hospital Nov. 30. The knee was greatly swollen and very painful, and fluctuation was distinct on either side of the lower third of the femur. The disease commenced about six years since, and was supposed to arise from injury; it had not however been so severe as to prevent use until for the last seven months. Puncture allowed the escape of several ounces of pus, but did not effect in any degree the distension of the knee, or abate very mnch, the severe pain experienced. Tonics, stimulants, and anodynes were freely administered; morphine becoming absolutely necessary to allay the pain, while full diet, stimulants, and tonics seemed to improve the general system in some degree. When first admitted, amputation was suggested as offering the only possible chance for recovery, and yet the uncertainties of the result were not concealed, and the prospect looked so disheartening as to discourage him from early accepting it.

At length the pain being severe, and the certainty of a fatal termination without operation so apparent, he became very anxious that it be made, and the operation was performed three months after admission, not however with expectation of doing more than relieve him of severe pain, offer him the only remaining hope, and thereby perhaps shorten his life by a few days, or even by a few weeks. His constitutional condition at the time of operation was not decidedly more unfavorable for recovery than on his admission, though the pain had greatly increased and he had grown to regard the removal of the diseased knee absolutely necessary to the continuance of life. The integument over the articulation was greatly distended, and without most careful examination it would have appeared certain to an observer that there was fluid accumulation in the synovial cavity of the joint and distinct fluctuation. This grew out of the infiltration and deposit about the joint, and was not regarded as fluctuation. After removal, dissection of the joint showed great disorganization of the ligaments and cartilages; the articular cartilages were thickened, softened, and easily separated from connection with the bone. The ligaments and tendons about the joint were involved in the disease, and from inflammatory action and deposit of lymph the whole structures had become pulpy, softened, friable, and easily broken down upon slight force. The bone was not very carefully examined, but was softened, roughened and easily cut or penetrated with a knife. The periostium was thickened and easily separated from the bone. This case presented all the characteristics of strumous disease, and yet no complications were present; the lungs were free from disease, and there was no indication of disease elsewhere. After operation, which was made February 21st, the system rallied completely from the shock, pulse remained firm, countenance cheerful, and in every respect the case looked hopefully as ever until a day or two before death, which took place March 3d, twelfth day after operation.

(To be continued.)

